# Previous chronic cerebral infarction is predictive for new cerebral ischemia after carotid endarterectomy

**DOI:** 10.1186/s13019-015-0367-x

**Published:** 2015-11-02

**Authors:** Mehmet Besir Akpinar, Veysel Sahin, Neslin Sahin, Ahmet Feyzi Abacilar, İlker Kiris, Ihsan Sami Uyar, Faik Fevzi Okur

**Affiliations:** 1 Department of Cardiovascular Surgery, Sifa University Faculty of Medicine, Fevzipasa Bulvari No: 172/2 Basmane Konak, 35240 Izmir, Turkey; 2 Department of Radiology, Sifa University Faculty of Medicine, Izmir, Turkey

**Keywords:** Stroke, Carotid endarterectomy, Silent cerebral ischemia, Chronic cerebral ischemia, Transient ischemic attack

## Abstract

**Background:**

The purpose of this study was to investigate the relation between preoperative chronic cerebral ischemia and postoperative new cerebral ischemia in patients undergoing carotid endarterectomy (CEA).

**Methods:**

We reviewed the diffusion weighted magnetic resonance images (DWI) of the 51 patients (37 men, mean age 68.8 ± 8.4 years) undergoing isolated CEA in the preoperative and early postoperative period. The number, anatomic location and the size of new ischemic lesions were recorded.

**Results:**

In the preoperative period, 28 (54.9 %) patients were symptomatic. There was chronic cerebral infarction in the preoperative DWI images of 17 patients (33.3 %). In the postoperative period, there was newly developed cerebral ischemia in postoperative DWI images of eight (15.7 %) patients. Six of the eight patients with newly developed cerebral ischemia had chronic cerebral infarction in their preoperative DWI images. The incidence of newly developed cerebral ischemia after CEA in patients with preoperative chronic cerebral ischemia was significantly higher than the incidence in patients without preoperative chronic cerebral ischemia (*p* = 0.01).

**Conclusion:**

The results of the present study suggest that preoperative chronic cerebral ischemia may aggravate postoperative newly developed cerebral ischemia in patients undergoing CEA.

## Background

Carotid Endarterectomy (CEA), first described in 1954 by Eastcott et al. [1], is a worldwide common procedure for the surgical treatment of carotid artery stenosis. The main objective of the CEA is to prevent or at least reduce the risk of future stroke. In concordance with this objective, well-accepted studies have documented that CEA ensured significantly better clinical outcome than medical therapy alone in both symptomatic and asymptomatic patients with > 70 % carotid artery stenosis [2–5].

However, the surgery to prevent stroke itself carries a risk of stroke. Most perioperative neurological complications are ischemic complications caused by hemodynamic hypoperfusion and emboli released from fragile plaque during the arterial dissection and shunting, cross-clamping and declamping period [6]. These perioperative neurological events can develop during or early after CEA both in symptomatic or asymptomatic patients. Many patients manifest silent cerebral ischemia without any clinical sign that only can be diagnosed with diffusion weighted magnetic resonance (DWI) [7–9]. DWI is one of the most sensitive tool to evaluate newly developed cerebral ischemia after CEA [10, 11].

Although mechanisms and accompanying factors during perioperative neurological complications have been intensely investigated, the relation between preoperative chronic cerebral ischemia and development of postoperative cerebral ischemia remains unclear. The purpose of the present study was to investigate the relation between preoperative chronic cerebral ischemia and postoperative newly developed cerebral ischemia in patients undergoing CEA. For this purpose, we reviewed the DWI images of the 51 patients undergoing CEA in the preoperative and early postoperative period.

## Methods

### Subjects

Between October 2012 and February 2013, 51 patients (37 male, mean age 68.8 ± 8.4) who underwent CEA operation in our clinic were included into the study. Patients with following conditions were not included in the study: non sinus cardiac rhythm, the presence of cardiac valvular disease, the presence of prosthesis inappropriate for MRI screening, history of cerebral operation, claustrophobia, disability and patients scheduled for concomitant open heart surgery.

Carotid artery stenosis was defined as symptomatic if ischemic stroke, transient ischemic attack (TIA) or retinal ischemia in the supply territory of the relevant carotid artery had occurred within the preceding 6 months [12]. The patients who did not have stroke, TIA or visual disturbance within the preceding 6 months were assumed as asymptomatic. Carotid artery stenosis was diagnosed by Duplex ultrasonography of carotid arteries in all patients. In case 60 % or greater stenosis in common or internal carotid artery was obtained by Duplex ultrasonography, the patient was referred to carotid arteriography screening by conventional angiography or computerized tomography (CT) angiography, including arch and selectively both common carotids.

Demographics of the patients, atherosclerotic risk factors, status of contralateral carotid arteries, and the site of the CEA are summarized in Table 1. We used carotid Doppler ultrasonography (USG) for the preoperative screening of the patients undergoing coronary artery bypass graft (CABG) surgery. All of the asymptomatic cases were determined with this method.

**Table 1:** Baseline characteristics of the patients

Characteristics	Asymptomatic patients (*n* = 23) (%)	Symptomatic patients (*n* = 28) (%)	Sum (*n* = 51) (%)
Demographics			
Age, years	68 ± 9.4	69 ± 7.6	68 ± 8.2
Male	18 (78.3 %)	19 (67.9 %)	37 (72.5 %)
Female	5 (21.7 %)	9 (32.1 %)	14 (27.5 %)
Site of the CEA			
Right	12 (52.2 %)	14 (50 %)	26 (51 %)
Left	11 (47.8 %)	14 (50 %)	25 (49 %)
Risk Factors			
Hypertension	13 (56.5 %)	17 (60.7 %)	30 (58.8 %)
Diabetes Mellitus	8 (34.8 %)	12 (42.8 %)	20 (39.2 %)
Hyperlipidemia	12 (52.2 %)	15 (53.6 %)	27 (52.1 %)
Ischemic heart disease requiring CABG	23 (45.1 %)	2 (3.9 %)	25 (49.0 %)
Contra Lateral Carotid Artery Stenosis			
< 50 %	17 (73.9 %)	19 (67.9 %)	36 (70.6 %)
50–70 %	4 (17.4 %)	1 (3.6 %)	5 (9.8 %)
≥ 70 %	2 (8.7 %)	3 (10.7 %)	5 (9.8 %)
Total Occlusion	–	5 (17.8 %)	5 (9.8 %)
Preoperative DWI Images			
Acute ischemia	3 (14.3 %)	2 (7.1 %)	5 (10 %)
Chronic ischemia	–	17 (60.7 %)	17 (33.3 %)
Postoperative DWI Images			
Acute ischemia	2 (8.7 %)	6 (21.4 %)	8 (15.7 %)
Acute hemorrhage	–	1 (3.6 %)	1 (2 %)

The numbers and the percentages of preoperative neurologic condition (symptomatic and asymptomatic cases) are summarized

The patients in the present study were without neurological disability and the time period between latest ischemic event and admission date was around 1 week.

Approval was obtained from the Clinical Research Ethics Committee of Sifa University (Sifa University Ethics Committee Clinical Trials Registration. date: 29.08.2012, number: B.30.2.SFU.00.50.500/49) and all subjects gave informed consent.

### Carotid artery ultrasound

Carotid Doppler Ultrasound was used to evaluate the Carotid artery disease. (Sonoline Antares, Siemens Medical Solutions, Erlargen, Germany, 7.5-MHz linear-array transducers). A ≥70 % ICA stenosis but less than near occlusion of the ICA is diagnosed when the ICA Peak Systolic Velocity (PSV) is greater than 230 cm/s and visible plaque and luminal narrowing are seen at gray-scale and color Doppler US. Additional criteria include ICA/CCA PSV ratio > 4 and ICA End Diastolic Velocity (EDV) > 100 cm/s. The higher the Doppler parameter lays above the threshold of 230 cm/s, the greater the likelihood of severe disease.

### Carotid arteriography

The amount of stenosis was calculated as in the NASCET study: minimum residual lumen at the point of maximum stenosis referenced to the diameter of the distal lumen of the internal carotid artery at the first point at which the arterial walls became parallel. We offered CEA operation to patients with ≥ 60 % ulcerated stenosis or ≥ 70 stenosis in common or internal carotid stenosis obtained by arteriography.

### Magnetic resonance screening and evaluation

MR imaging was performed using a 12-channel phased array head coil on a 1.5 T clinical scanner (Espree; Siemens, Erlangen, Germany). In addition to conventional MR sequences including fluid-attenuated inversion recovery (FLAIR), T1-weighted imaging and T2-weighted imaging, DWI and SWI sequences were also obtained. DWI was performed with a spin-echo-type EPI sequence: TR, 4200 ms; TE, 114 ms; b values of 0, 500 and 1000 s/mm^2^; FOV 230 × 230 mm; matrix size 256 × 256; section thickness, 5 mm. The SWI consisted of a long TE (TR/TE 49/40 ms, flip angle 20°) fully flow-compensated 3D FLASH sequence with a slice thickness of 2 mm, FOV 230 × 230 mm, and matrix size 320 × 320.

MR imaging with DWI and SWI was prospectively performed a day before and in the time period between 24 and 48 h after CEA. MRI sequences were used to detect any ischemic lesions before and after CEA. On DWI, any new hyperintense area - regardless of size - the brain tissue was considered a sign of new ischemic lesions; on SWI, new hypointense signal intensity changes were interpreted as a sign of acute hemorrhage.

High signal intensity on T2-weighted and low signal intensity on T1-weighted images without diffusion restriction at one or more vascular territories or at border-zones were defined as ‘chronic ischemia’. CEA timing for the symptomatic patients was one week after the occurrence of last symptom. The definition of ‘chronic ischemia’ has been done based on features of DWI images. Therefore, timing of the symptoms such as stroke or TIA had not been taken into consideration during this definition.

The number, anatomic location and the size of ischemic and hemorrhagic lesions were recorded. All the images were evaluated by an experienced radiologist (NS).

### Operative procedure

All of the patients were operated on under general anesthesia. Systolic blood pressure was maintained at 140–150 mmHg during carotid clamping. Eight mg of dexamethasone was administered before the application and after the removal of the cross clamp.

The carotid arteries were explored through a longitudinal incision parallel to the medial aspect of the sternocleidomastoid muscle. The common, internal and external carotid arteries were explored. Following the intravenous administration of 200 unit/kg heparin, CEA performed with eversion technique. The heparin was then reversed with protamine, and the patients were transferred to the intensive care unit, extubated and followed up.

Antiplatelet therapy was continued during the preoperative and postoperative period, and all of the patients were prescribed acetylsalicylic acid (150 mg oral/daily).

### Statistical analysis

The demographic parameters of patients (gender, diabetes, hypertension, hyperlipidemia), the percentage of carotid stenosis, operative indication (symptomatic, asymptomatic) and the surgical sites (left, right) were evaluated. The preexisting ischemias in the DWI were determined. We examined whether all of these factors affected the development of new cerebral ischemia after CEA. Chi-squared Fisher Exact test was used for the statistical analysis. Statistical significance was set at a *P*-value < 0.05.

## Results

The mean age of the 51 studied patients was 68.8 ± 8.4 years (range 46–82). There were 37 men (72.5 %) and 14 women (27.5 %). Arterial hypertension (58.8 %) and hyperlipidemia (52.9 %) were the most frequent risk factors, followed by diabetes mellitus (39.2 %). Of the 51 carotid artery stenosis cases, 28 (54.9 %) were symptomatic (15 with TIA or amaurosis fugax and 13 with stroke) and 23 (45.1 %) were asymptomatic preoperatively. Twenty-six (51 %) patients operated from the right carotid artery, while 25 (49 %) were from the left. The contralateral internal carotid artery was occluded in 5 patients (9.8 %) and over the 70 % degree stenosis in 5 (9.8 %). All these data are summarized in Table 1. Evidence of coronary artery disease requiring CABG was present in 25 patients, and these cases were referred to coronary bypass surgery a few days after CEA. The mean operative time was 72 ± 18 min., and mean cross clamp time was 13.2 ± 5.3 min. In eight patients (15.7 %), new cerebral ischemia was detected in DWI images within 24–48 h after CEA.

On pre-operative DWI images, acute and chronic cerebral ischemia was observed in 5 and 17 cases, respectively (Table 1).

**Fig. 1:**
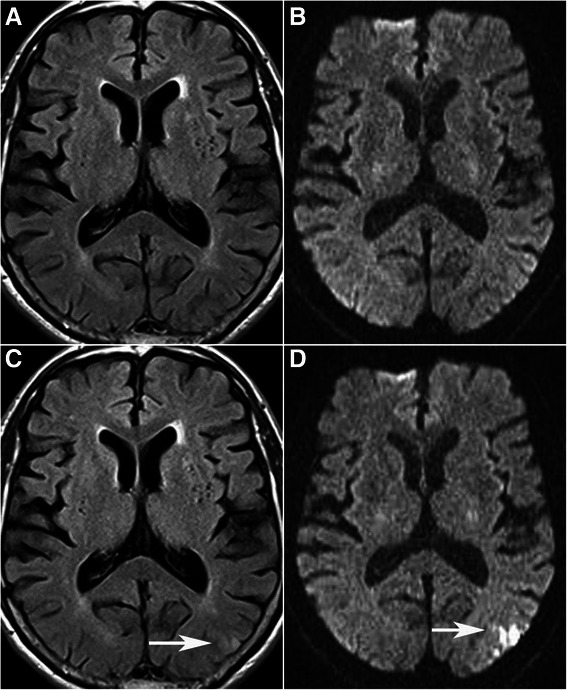
Seventy-four-year-old man with 70 % stenosis of left ICA. Preoperative (**a**) FLAIR and DWI (**b**) images show no abnormality. Postoperative FLAIR (**c**) and DWI (**d**) images demonstrate new left parietal small acute ischemic lesion (*white arrow*) which is not seen on preoperative images (**a**, **b**)

On post-operative DWI images; new cerebral ischemia was observed in 8 (15.7 %) and a new hypointense signal (hemorrhage) in 1 (3.6 %) case. Six of the 8 patients with new cerebral ischemia were the patients that had chronic infarction in their pre-operative DWI images. Most of the identified changes of the new signals were on the same side as the operation, and also near the chronic ischemic area (Fig. 1). The patient which the hemorrhage detected was one of the pre-operative chronic ischemia cases, and this patient developed a 4-mm diameter hemorrhage in the same location of ischemia after CEA. The locations, diameters and volumes of the lesions are summarized in Table 2.

**Table 2:** The size and location of postoperative DWI ischemic lesions, preoperative neurologic status and the level of contralateral carotid artery stenosis

Patients (Names initials)	Preoperative symptoms	Site of the CEA	Pre operative DWI images	New ischemic areas on DWI (cm^3^)	Contralateral carotid artery stenosis (%)
ME	Asymptomatic	Left	Normal	Left occipital cortical 0.2 cm^3^	60
NY	Asymptomatic	Right	Normal	Right parietooccipital cortical 0.55 + 0.06 + 0.26 cm^3^	0
HB	TIA	Left	Left frontal chronic ischemia	Left frontal cortical 0.026 cm^3^	20
HD	TIA	Right	Right occipital- right parietal chronic ischemia	Right posterior parietal cortical 0.016 + 0.022 cm^3^	70
MB	TIA	Right	Right parietal chronic ischemia	Right frontoparietal 0.011 cm^3^	0
MA	Stroke	Right	Bilateral parietal chronic ischemia	Right frontoparietal 2.88 + 0.034 cm^3^	70
NG	Stroke	Left	Left occipital chronic ischemia	Left occipital 0.04 cm^3^	0
HE	Stroke	Right	Left occipital chronic İschemia	Left parietooccipital 6.48 cm^3^	100
FB	TIA	Left	Left parietal chronic ischemia	Thalamic hemorrhage 0.025 cm^3^	0

The last case on the list suffered cerebral hemorrhage

*CEA* Carotid endarterectomy, *TIA* Transient ischemic attack, *DWI* Diffusion-weighted MRI

**Fig. 2:**
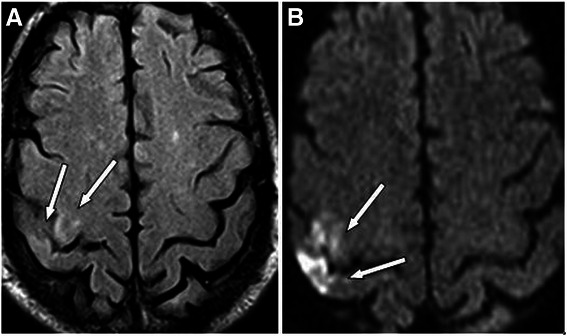
Sixty-eight-year old man with 80 % stenosis of right ICA. Preoperative DWI image shows right occipital chronic ischemia (**a**). Postoperative DWI image demonstrate new right parietooccipital ischemic lesion (**b**) (White arrows)

Chronic cerebral infarction was found in the preoperative DWI images of 17 patients (33.3 %). DWI images showed that 6 of these 17 patients (35.3 %) experienced new silent cerebral ischemia in the post-operative period (Table 2) (Fig. 2). On the other hand, in the patients without chronic cerebral infarction (n: 34), we discovered 2 new postoperative cerebral ischemia (5.9 %) in their DWI images. The incidence of newly developed cerebral ischemia after CEA in patients with preoperative chronic cerebral ischemia was significantly higher than the incidence in patients without preoperative chronic cerebral ischemia (*p* = 0.01). The statistical analysis of chronic cerebral ischemia and newly developed cerebral ischemia are shown in Table 3.

**Table 3:** Statistical table of risk factors for new cerebral ischemia after carotid endarterectomy

Characteristic	No. of patients	No. of patients with postoperative new ischemia	*P*-value
Gender			
Male	37	6	
Female	14	2	1.0
Hypertension			
Yes	30	5	
No	21	3	1.0
Diabetes Mellitus			
Yes	20	3	
No	31	5	1.0
Hyperlipidemia			
Yes	27	4	
No	24	4	1.0
Contralateral Carotid Stenosis			
Occlusion or ≥ 70 %	10	3	
Normal to moderate	41	5	0.17
Preoperative neurologic status			
Asymptomatic	23	2	
Symptomatic	28	6	0.26
The Site of the CEA			
Right	26	4	
Left	25	3	1.0
Preoperative Brain Images			
Chronic infarction	17	6	
No chronic infarction	34	2	***0.01***

There is a significant positive correlation between preoperative chronic infarction and new postoperative cerebral ischemia (*p* = .01)

The mean cross clamp time was 12 ± 3 min in 8 patients in whom we determined new ischemia after CEA. There were no significant differences between the patients for new ischemia by cross clamp time, contralateral carotid artery stenosis rate, and demographical information (*p* > 0.05, Table 3).

None of the patients experienced hematoma or infection on the operation area. We detected ipsilateral peripheral nerve damage (lingual deviation) in 1 patient.

One patient suffered ipsilateral hemiparesis for two hours after CEA. And this ischemic symptom disappeared after 6 h from CEA. The remaining new ischemias were silent cerebral ischemia.

## Discussion

The results of the present study suggest that preoperative chronic cerebral ischemia may aggravate postoperative newly developed cerebral ischemia in patients undergoing CEA. The main finding of the study supporting this suggestion is the incidence of new cerebral ischemia in patients with preoperative chronic cerebral ischemia was significantly higher than the incidence in patients without preoperative chronic cerebral ischemia.

Cerebral infarction is a focal brain necrosis due to ischemia that affects all tissue elements of the brain. Most of the cerebral infarcts are caused by atherosclerosis of cerebral arteries, alone or with superimposed thrombosis. The risk factors for stroke include age, hypertension, diabetes mellitus, smoking, prior cardiovascular disease (coronary heart disease, cardiac failure or intermittent claudication), atrial fibrillation and left ventricular hypertrophy by electrocardiogram [13]. The patients whom have 70–99 % stenosis in extracranial carotid arteries has 16.8–21.9 % risk of stroke rate per yearly [5]. Concordance with this report, we obtained previous cerebral ischemia in 22 of 51 patients in the preoperative period. The majority of the patients (17 of 22 patients) with previous cerebral ischemia had chronicle cerebral lesions.

DWI is used for detection of cerebral acute ischemia. DWI detects when H_2_O (hydrogen protons) are unable to diffuse freely. With cytotoxic edema, the H_2_O that normally surrounds the cells cannot diffuse as easily and becomes “restricted.” DWI is very sensitive at detecting this change. Than the images of DWI becomes hyperintense (bright/positive) within minutes of acute ischemia [14, 15].

The symptoms and signs of TIA or stroke are indicators of cerebrovascular disease of the brain. The sensitivity rate of DWI for symptomatic cerebral ischemia is over 90 % [16]. In our study, we used DWI as a sensitive diagnostic method to obtain acute cerebral ischemia. In addition, according to preoperative DWI images of the cases, we detected chronic cerebral ischemia in 60 % of symptomatic patients. However, the results of neuropathological studies have shown that vascular disease manifesting as infarcts can result in injury to the brain in the absence of transient ischemic attack or stroke. These rates were determined 12–20 % in Framingham and Rotterdam Scan Study studies, respectively [17, 18]. In our study we did not detect any cerebral infarction in any asymptomatic case.

There are different surgical techniques for CEA including transvers or longitudinal arteriotomy, eversion or conventional techniques with carotid shunt usage [19]. It has been reported that eversion CEA has a lower restenosis rate than conventional CEA closure techniques and thus superior long-term durability. Today, eversion CEA is one of the most preferred technique with a postoperative stroke rate between 0.6 and 2.5 % [20–22]. In this study all of the cases were operated with eversion CEA technique. Thus, we did not use carotid shunt for any patient due to the nature of the surgical technique.

Perioperative reduction of cerebral blood flow due to intraoperative clamping of the carotid artery and subsequent neuronal damage is reported to be the main cause of postoperative neurologic complications following CEA. Although its pathophysiology remains incompletely understood, micro emboli or subclinical micro infarcts are accused of being additional risk factors [23–25]. Vanmaele [26] noted a shorter cross-clamp time with eversion CEA. In our study, the mean carotid clamping time was also relatively short (13.2 ± 5.3 min.) In addition, carotid cross clamping time of the patients with postoperative newly developed cerebral ischemia was not significantly different than of patients without cerebral ischemia.

In our study, there were contralateral carotid occlusion and severe (70 % or higher) carotid artery stenosis in five and five patients, respectively. New cerebral ischemia developed in one patient with contralateral carotid occlusion and in two patients with contralateral severe carotid artery stenosis. Thus, we did not find a significant increment in the risk of development of new cerebral ischemia in patients with severe stenosis or occlusion in contralateral carotid artery stenosis.

Carotid artery disease is one of the etiological factors in the pathophysiology of post-CABG stroke [27]. Surgical management of the patients with coronary artery disease requiring CABG and carotid artery stenosis requiring CEA is still in debate. In our clinic, in patients with a stable coronary artery status, we prefer a staged procedure as first CEA-later CABG approach. In case critical proximal left anterior descending (LAD) or left main coronary artery stenosis exist, we prefer concomitant CABG and CEA operation. In the present study, the patients who underwent staged procedure but not concomitant procedure were included into the study.

There are different rates in the literature for the incidence of new or silence cerebral ischemia after CEA, including 0.2–15 % [28, 29]. Silent brain infarctions are ischemic silent radiologic abnormalities that act as predictors of subsequent strokes and these lesions are associated with subtle deficits in physical and cognitive function that commonly go unnoticed [30, 31]. The presence of silent infarcts more than doubles the risk of subsequent stroke and dementia [32].

In our clinique postoperative symptomatic stroke rate is under 2 % after CEA [22]. In this study, we found 8 new ischemic images on DWI which 1 of them symptomatic (1.9 %), 7 of them asymptomatic –silence- ischemia (13.7 %) after CEA. According to the diameter of the ischemic areas, the largest one was 6.48 cm^3^ (24-mm diameter), while the remaining cases were smaller than 5 mm in diameter. The symptomatic patient experienced ipsilateral hemiparesis had 6.48 cm^3^ ischemia in parietooccipital region. We also identified 1 cerebral hemorrhage after CEA. In this case cerebral hemorrhage was at thalamic region.

The weakest sides of the artery capillary system of the brain are also the sides that are most sensitive for hypoperfusion. In these areas, the presence of ‘Watershed-border zone infarction’ decreases perfusion pressure and increases the ischemia risk in the border zones [33]. The ischemic penumbra is described as the viable brain tissue adjacent to the area of ischemic infarction [34]. This area is more susceptible to ischemia than healthy brain tissue.

Incomplete and previous infarcts may also be the basis for silent brain ischemia. The effect of chronic cerebral infarctions on silent ischemia is not so clear. We couldn’t find any literature about the effect of previous chronic cerebral infarctions on new cerebral ischemia development after CEA. It is thought that symptoms are absent because of the ischemic precondition [35].

In large series in the literature symptomatic carotid artery disease is accepted with an increased risk factor for stroke or death [36, 37]. Our study showed no significant difference in terms of the emerging cerebral ischemia in preoperative symptomatic and asymptomatic patients. All patients with chronic cerebral ischemia were symptomatic preoperatively. However, there was no chronic ischemia in all preoperative symptomatic cases. Accordingly, when defining the preoperative risk factors ‘existence of chronic ischemic areas’ may be an additional definition of risk for new-silent cerebral ischemia. Further studies are needed for this.

However, with a small sample size, caution must be applied when interpreting our results. All cases of new cerebral ischemia occurred in patients with previous chronic cerebral infarction and were also on the same side as the infarction. It can thus be suggested that chronic cerebral infarction makes the brain vulnerable to new ischemia.

In our patients, ischemia was similar to cortical and watershed infarcts that appeared on the sides of previous infarctions. In a case (one of the contralateral carotid occlusion cases) had chronic brain ischemia in occluded site on preoperative MRI image. In this case, postoperative DWI images showed new ischemic lesion on the contralateral (occluded) site, not on the operation site. This case also strengthened the idea that hypoperfusion is responsible for the ischemia. Therefore, especially in patients who have old infarcts, the brain is more susceptible to hypoperfusion. However, more research on this topic needs to be conducted.

## Conclusion

In conclusion, the results of the present study suggest that preoperative chronic cerebral ischemia may aggravate postoperative newly developed cerebral ischemia in patients undergoing CEA. Regardless of whether they were symptomatic or not, the patients whose preoperative DWI images revealed chronic ischemia had much higher incidence of silent ischemia development after CEA than the patients who did not. Hence, it could conceivably be hypothesized that previous cerebral infarction was predictive for new silent cerebral ischemia after CEA. However, the mechanisms of cerebral ischemia are very complex and prospective clinical trials with large number of patients are needed to make more clear conclusions in the future.

## Abbreviations

CEA: Carotid endarterectomy
DWI: Diffusion weighted magnetic resonance
SWI: Susceptibility weighted imaging
MRI: Magnetic resonance imaging
TIA: Transient ischemic attack
CT: Computerized tomography
USG: Doppler ultrasonography
CABG: Coronary artery bypass graft
ICA: Internal carotid artery
CCA: Common carotid artery
PSV: Peak systolic velocity
EDV: End diastolic velocity
